# Investigation of antibacterial and photocatalytic efficiency of green ZnO nanoparticles that synthesized with *Celosia Cristata* flower extract

**DOI:** 10.3906/kim-2007-10

**Published:** 2021-09-01

**Authors:** Mahmure ÜSTÜN ÖZGÜR, Özgür DUYGULU, Melda ALTIKATOĞLU YAPAÖZ

**Affiliations:** 1 Department of Chemistry, Faculty of Arts and Science, Yıldız Technical University, İstanbul, Turkey; 2 TÜBİTAK MAM Materials Institute, Gebze, Kocaeli, Turkey

**Keywords:** Zinc oxide nanoparticles, green synthesis, *Amaranth*, *Celosia cristata L*., antibacterial activity, photo catalysis, Basic violet 39, Basic yellow 28

## Abstract

Increasing interest in green chemistry has led scientists to an environmentally friendly nanoparticle synthesis approach that has many advantages, such as simple, affordable and versatility for a wide range of commercial production. In this study, green synthesis of zinc oxide nanoparticles (ZnO NPs), which is widely researched in the field of nanotechnology, was performed under different conditions (volume ratio of CC flower extract to Zn(CH_3_COO)_2_ solution, time, pH and temperature) using the aqueous extract of *Amarant* (*Celosia cristata L*., CC, cockscome) plant flowers. Produced ZnO NPs were characterized by UV-Vis absorption spectroscopy, Fourier transform infrared spectroscopy (FTIR), high-resolution transmission electron microscopy (HR-TEM) and scanning electron microscopy (SEM) analysis. The characteristic absorption peak seen at λ_max_: 364 nm in the UV-Vis absorption spectrum and the band seen at 381 cm^−1^ in the FTIR spectrum indicate that ZnO NPs were synthesized. TEM image also confirmed the formation of nanoparticles. The average size of nanoparticles is approximately 22–27 nm and the shape of the ZnO NPs as nearly spherical. The effect of different calcination temperatures (100, 200, 300, 400, and 500 °C) on the size of ZnO NPs was investigated and it was observed that the particle size decreased as the calcination temperature increased. ZnO NPs were also used as photo catalyst for removal of basic yellow28 (BY28) and basic violet39 (BV39) dyestuffs which are used in textile industry and ecologically toxic. The decolorization efficiency was found 95%–100% and 62% respectively when the BV39 and BY28 dyestuffs were exposed to UV light for 160 min. Antibacterial activity of ZnO NPs produced with different amounts of CC flower extract and calcined at different temperatures (100, 200, 300, 400, and 500 °C) was investigated using modified disc diffusion method. Produced ZnO NPs displayed antibacterial activity against *Staphylococcus aureus* and *Escherichia coli* bacterial strains and were more effective against gram-positive pathogens. The findings displayed that the antibacterial activity of ZnO NPs is related to the particle size. This new environmentally friendly synthesis approach is a suitable technique for large-scale commercial production and can be considered as an alternative to chemical methods.

## 1. Introduction

The rapid development of studies on nanotechnology has increased the importance of nanoparticles used in many fields. Zinc oxide (ZnO) is nontoxic and compatible with the skin; with its properties such as UV reducing effect, it is also used in the field of human health. The compatibility of ZnO NPs with liquid and organic solvents allows for the participation of many materials processes, has a wide band range, and allows the use of nanoscale optoelectronics and piezoelectric-nanogenerators in biotechnology [1]. For years, the synthesis of nanosized ZnO powders has been devised by different methods such as direct precipitation and coprecipitation [2, 3], hydrothermal synthesis [4], spray pyrolysis [5], sol-gel combustion process [6], vapor transport processing [7] and electrochemical growth [8]. Due to the environmental and biological risks of chemicals, there has been increasing interest in environmentally friendly, inexpensive, biomedical and drug-compatible techniques in recent years [9]. Biosynthesis of metal oxide nanoparticles using different parts of the plant is a novel alternative that does not produce toxic chemicals [10]. Using plant extract to produce metal oxide is an important advantage due to the production of bioactive molecules that reduce metal ions [11]. Often, the phytochemicals in different parts of plants (roots, leaves, flowers and fruits, etc.) such as phenols, flavonoids, amino acids, aldehydes, alkaloids, ketones, terpenoids, vitamins, proteins and amides, can take part in the reduction, production and stabilization of metal and metal oxide nanoparticles [12,13]. It is reported that plant concentrates containing these phytochemical shave been used for the green synthesis of ZnO nanoparticles. Recently, remarkable comprehensive studies have been conducted on the green synthesis and application areas of ZnONPs [14,15]. Some of the plants used in these studies are *Camellia Sinensis* [16], *Hisbiscus rosa-sinensis* [17], *Psidium gujava* [18], *Daucus carota* [19], *Brassica oleracea L. var. italica* (Broccoli) [20]. The reaction conditions used in the production of ZnO NPs using different plants and some data of the obtained particles are given in Table 1. In order to make possible the nanoparticle synthesis with the green methods to contest with the chemical techniques, quicker synthesis is needed. This study was conducted on a more environmentally friendly and fast production of ZnO NPs. The exact reduction of Zn^2+^ ions occurred in around 2 h. As seen in Table 1, the time required for green synthesis is shorter than the other studies. In addition, when compared with other studies in Table 1, synthesis temperature (40 °C) is lower than the others and very small (approximately 22–27 nm) particle-sized spherical ZnO NPs were obtained in our study and these small nanoparticles remained stable 11 months at room temperature storage. The method developed with these features can be an alternative to these studies.

Today, the depletion of worldwide energy resources and environmental pollution are the most important problems that threaten human life. Industrial wastewater containing dyestuffs is the primary reason for the pollution of water resources. As well as toxic these dyestuffs that threaten the ecosystem and human health are resistant to sunlight and temperature. In the dyeing process in the textile industry, when a large amount of colored wastewater was given to receiving water environments, it decreases the light transmittance of the water environment and thus toxic and carcinogenic compounds may be formed from the reactions such as oxidation in aquatic environments and degradation of dyestuffs. Therefore, the treatment of wastewater in the textile industry is gaining great importance from an ecological perspective [32]. Many physical and chemical methods are used to treat the dyestuffs present in the industry before discharge to the environment and these methods are improved with the developing technology. Within the recent scientific studies for the solution of these problems, photoelectrochemical hydrogen production involving the photocatalytic decomposition of toxic organic substances in water and the use of renewable energy sources draw attention. All of these studies are based on the heterogeneous photocatalysis process using semiconductor materials that can be activated when they absorb the necessary energy in the form of radiation [33]. ZnO has attracted great attention in recent years among photocatalyst semiconductors due to its new technology in environmental pollution. Amaranthus (Amaranthus spp) is a generic term for Amaranthus genus plants in the Amaranthaceae family, covering about 60–70 plant species. *Celosia Cristata* (CC) is a member of the genus Celosia of the Amaranthaceae family and is often referred to as cockscombs in Turkey. Methanol extract of this plant mainly contains phenols, flavonoids, carbohydrates, alkaloids, tannins, glycosides, etc., and phytochemical compounds in plant flowers are used for the treatment of hemorrhoids and diarrhea [34]. It is stated that grains of *Amaranth* plant flowers can be used in increasing the nutritional value of various foods with amino acids and high-quality protein content. In addition to antihypertensive properties of the cholesterol-lowering effect is pointed out [35]. Various properties such as antioxidant, antiviral, and anthelmintic effects have also been investigated [36, 37].

To the best of our knowledge, there is no reported study in the literature on the synthesis of metal oxide nanoparticles by means of green chemistry using *Celosia Cristata* flowers (CCF). In this study, using the aqueous extract of the *Celosia Cristata* flowers for the first time, nanoparticles were obtained by both economical, fast and environmentally friendly biosynthesis methods. This new environmentally friendly synthesis approach is a useful technique suitable for large-scale commercial production.

ZnO NPs have excellent antimicrobial, antibacterial, and fabulous UV blocking properties. ZnO, with its antibacterial effect, is used in medicine, cosmetics, food, textiles, high-quality paint, sunscreen and antiultraviolet fabric, smart textile products, wastewater treatment, and many other areas. Because of ZnO’s robust UV absorption, they are increasingly utilized in individual care products such as sunscreens and cosmetics. Nano-ZnO allows zinc to be ingested more effectively by the body. Therefore, nano-ZnO is broadly utilized in the food, pharmaceutical, and food packaging industries. Compared to other metal oxide nanoparticles, ZnO NPs, which are relatively inexpensive and comparatively less poisonous, are considered relatively compatible in biomedical and also food, cosmetic, and pharmaceutical industries if their particle sizes are smaller than 100 nm. Due to these features, interest in the use of ZnO NPs in these areas is gradually increasing. In this study, it was aimed to synthesize ZnO NPs, which can be used in these areas, smaller than 100 nm and expected to show antibacterial properties.

In this research, an aqueous extract of CC flowers (CCF) was used in the synthesis of ZnO NPs by the green method. We researched the effects of reaction conditions such as volume ratio of Zn-Ac solution to CCF extract, pH, reaction temperature and time on the particle size and stability of the produced ZnO NPs. ZnO NPs were characterized by UV-Vis, FTIR, HR-TEM and SEM techniques. In this study, the removal of BV39 and BY28 from aqueous solution was investigated using photocatalyst combinations (amount of ZnO NPs/UV light/time). The antibacterial activity of ZnO NPs produced with different amounts of CCF extract and calcined at different temperatures was also investigated using the modified disc diffusion method.

## 2. Materials and methods

### 2.1. Materials and instrumentation

**Figure 1:**
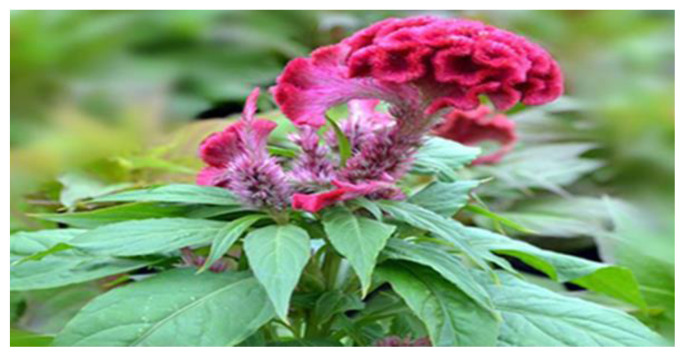
Digital photograph of Celosia cristata L. plant.

Zinc acetate dehydrate, Zn[CH_3_(COO)]_2._2H_2_O, was purchased from Sigma-Aldrich. Five grams of zinc acetate dehydrate (Zn-Ac) was dissolved with 500 mL distilled water first by stirring on a magnetic stirrer for 15 min and then incubated in an ultrasonic bath for 15 min at 30 °C. Ethyl alcohol and ammonia were of analytical grade (Sigma, St.Louis, MO, USA). Stock dyestuff solutions at a concentration of 100 μg/mL were prepared with distilled water. The bacterial cultures were *E. coli* (ATCC 25922) and *S. aureus* (ATCC 43300). All organisms were maintained on specific media slants at 4 °C and revived prior to use. Log phase bacterial inoculums (108 cfu/mL) were standardized against MacFarland’s standard. Flowers of *Celosia cristata L*. plant used in this study (Figure 1) were collected from Gürpınar, İstanbul and fresh flowers of the plant incised into small pieces.

All spectral measurements and treatment of data were carried out on a double beam Shimadzu UV-1800 spectrophotometer (Japan) and UV-probe 2.35 software was used to process the absorption and derivative spectra. All spectra recorded over the range 300–650 nm. High-resolution transmission electron microscopy studies were performed with JEOL JEM 2100 HRTEM at 200 kV. Images were taken by Gatan Model 833 Orius SC200D CCD camera. Carbon support film-coated copper TEM grids (Electron Microscopy Sciences, CF200-Cu, 200 mesh) were used after sonication of particles with ethanol. SEM studies were performed both by JEOL JSM 6335F FEG-SEM and JEOL JSM 6510-LV SEM. Investigations were performed at 15 kV of acceleration voltage after dropping a few drops of particle ethanol mixture on carbon tape. For functional group analysis, FTIR spectra of CC flower extract and produced ZnO NPs were recorded with a Perkin Elmer FTIR spectrophotometer.

### 2.2. Preparation of aqueous extract of CC flowers

The fresh flowers of CC were chopped into small pieces. For extraction of the biogenic-compounds of flowers, about 50 g of flowers was weighed and transferred into a 500-mL beaker containing 250 mL distilled water. Then, the reaction mixture was stirred at 250 rpm at room temperature for 3 h. The extraction process was repeated with 200 mL distilled water and the extracts were combined. Plant extract was centrifuged at 5000 rpm for 10 min and then filtered through Whatman filter paper (No. 2) to remove any suspended particles. Finally, a clear filtrate was obtained, and the total volume of the filtrate was diluted to 500 mL. Filtrate was stored in the dark at +4°C to be used within one month for further experiments.

### 2.3. Production of ZnO NPs

**Figure 2:**
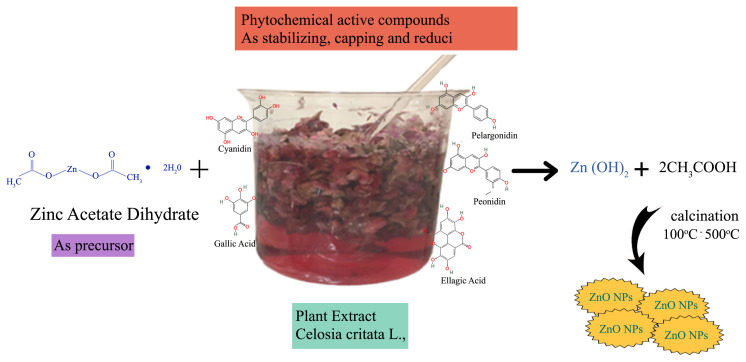
Suggested reaction mechanism for the green synthesis of ZnO NPs [38].

For the synthesis of ZnO NPs, 40 mL of CCF extract (10%, g/mL) was transferred into a 250-mL erlenmeyer flask and 50 mL of Zn-Ac solution was added drop by drop under constant stirring (this process was completed in about one hour). The pH of the prepared solution was adjusted to pH 10 with ammonia solution and continuously stirred at 40 °C for 2 h to complete the reaction. During the reaction period, the color of the solution changed from slightly red to white, which indicates the formation of ZnO NPs. The proposed reaction mechanism for the green synthesis of ZnO NPs is given in Figure 2.

**Figure 3:**
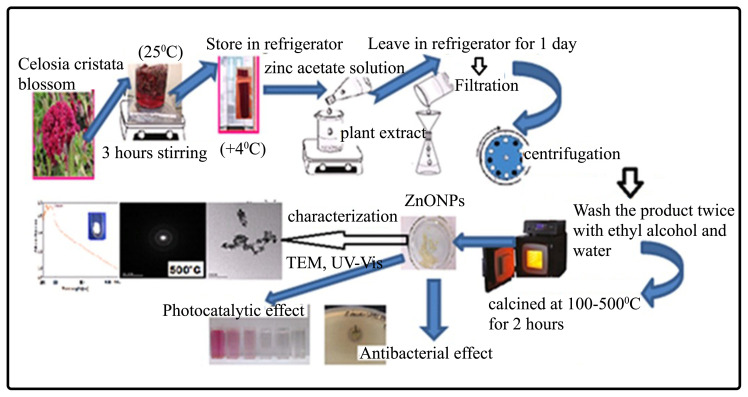
Representative view of the green synthesis stages, characterization and usage areas of ZnO NPs.

The resulting colloidal solution was allowed to stand in the refrigerator for 12 h. Obtained white suspension was centrifuged twice at 5000 rpm for 15 min. The clear supernatant was then separated. Solid ZnO NPs were washed two times, first with distilled water and then with ethyl alcohol thoroughly, and purified by centrifugation. Finally, produced ZnO NPs were calcined at different temperatures ranging from 100 °C to 500 °C for 2 h in a muffle furnace to transform the residual Zn(OH)_2_ into ZnO and powders were maintained at room temperature in a desiccator for further experiments. For the spectrophotometric measurements, 0.01 g of the ZnO NPs, calcined at different temperatures, were weighed separately and dissolved in 10 mL of ethyl alcohol. These solutions were diluted 1/10 with distilled water and the UV-Vis spectra of the obtained solutions were recorded in the wavelength range of 300–500 nm. The green synthesis stages, characterization and usage areas of the ZnO NPs produced in this study are given in Figure 3.

### 2.4. Effect of different parameters on ZnO NPs synthesis

Different parameters which had been identified as factors affecting the synthesis yield of ZnO NPs including reaction temperature, pH, volume ratio of Zn-Ac solution to CCF extract and reaction time were studied and optimized. ZnO NPs formation was monitored by UV-Vis spectroscopy. A constant concentration of Zn-Ac (50 mL, 1%) was used as a precursor. The effect of time on the synthesis reaction was examined under constant experimental conditions (temperature: 60 °C, the volume ratio of Zn-Ac solution to CCF extract: 1/0.8, pH 10). The reaction mixture was continuously mixed using a magnetic stirrer and the pH of the medium was adjusted to pH 10 by adding ammonia solution. To optimize the time required to terminate the reaction, the reaction was observed by taking the absorption spectra of the samples, which were taken from the reaction medium at 30 min intervals for 120 min. During the reaction period, the color of the solution was also observed and changed from slightly red to white, which indicates the formation of ZnO NPs. The time required to terminate the reaction was found to be 2 h.

To investigate the effect of the amount of CCF extract on the synthesis reaction, the volume of Zn-Ac solution was taken as 50 mL. A total of 50 mL of Zn-Ac solution was added drop by drop in 10, 20, 30, 40, 50, and 60 mL of CCF extract in about 60 min (the volume ratio of Zn-Ac solution to CCF extract: 5.0, 2.5, 1.66, 1.25, 1.0, and 0.8, respectively). The reaction temperature was adjusted to 60 °C, the pH value of the environment to pH 10, and the reaction mixture was stirred for further 2 h using a magnetic stirrer to form ZnO NPs. It was observed that the optimum volume ratio was 1.25 (50 mL Zn-Ac solution/40 mL CCF extract).

The effect of the reaction temperature on the synthesis reaction was carried out by repeated experiments under constant experimental conditions (reaction time: 2 h, volume ratio of Zn-Ac solution to CCF extract: 1.25 and pH: 10) at three different temperatures (40 °C, 60 °C, and 80 °C). The temperature of the reaction medium was maintained at 40, 60, and 80 °C using a water bath on a magnetic stirrer. The optimum temperature was determined to be 40 °C.

The effect of the pH value of the medium on the synthesis reaction was investigated for pH 6, 8, 10 and 12 under optimum conditions (reaction time: 2 h, volume ratio of Zn-Ac solution to CCF extract: 1.25 and temperature: 40 °C). The procedure applied in the biosynthesis section (subsection 2.3) was repeated for 4 different pH values. pH 10 was chosen as the most suitable medium pH value. Obtained pale white precipitate was dried in a hot air oven at 80 °C for 2 h to yield ZnO NPs. Powdered ZnO NPs were resuspended in an ultrasonic bath for 15–20 min to receive absorption spectra.

### 2.5. Use of produced ZnO NPs as photocatalyst

ZnO NPs, produced under optimum conditions and calcined at 300 °C for 2 h, were used as a photocatalyst to remove of BY28 and BV39 dyestuffs which are used in the textile industry and are ecologically toxic. Properties of the dyestuffs used in this study are given in Table 2. The photocatalytic efficiency of the ZnO NPs was assessed by decolorization of BV39 and BY28 dye solutions under UV light. Within the experiments, 10 ppm dye solution and different amounts of catalyst (0.01–0.10 g ZnO NPs) were used. The total volume of each reaction solution was kept up at 30 mL. A total of 100 mL beaker was utilized as a reactor equipped with a magnetic stirrer to ensure steady stirring of the solution in the reactor. The acidity of the solution was checked before and after the photo catalysis process. The reaction mixture was stirred in a dark environment for 60 min to achieve the liquid/solid phase balance and afterward, was stirred under UV light irradiation (wavelength: 254 nm, power: 32 W) for 160 min. While dyestuff solutions containing different amounts (0.02–0.10 g) of ZnO NP were stirred under UV light for 160 min, the absorption and first derivative absorption spectra of the samples, taken at different time intervals (at 45th, 90th, and 160th min), were recorded in the wavelength range from 300 to 650 nm. After 160 min, the solutions were filtered and first derivative absorption spectra were taken again. Decolorization of the solutions was calculated from the measured derivative absorbance values and the relevant calibration curves.

### 2.6. Antibacterial activity studies

In order to research the antibacterial efficiency of the ZnO NPs on selected bacteria, the agar well diffusion method was used [39]. ZnO NPs, synthesized at different volume ratios of Zn-Ac solution to CCF extract and calcined at different temperatures, were used to investigate the antibacterial activity. To determine the antibacterial efficiency, *Staphylococcus aureus* from the gram+ bacteria and *Escherichia coli* standard strains from the gram-bacteria were used. The stock cultures of the bacteria were seeded on nutrient agar to be resuspended and incubated at 37 °C for 16–18 h. The prepared bacterial suspensions were seeded into the Mueller Hinton agar environment with the help of a swab. A total of 100 μL of CCF extract and ZnO NP colloidal solutions were added to wells drilled in a solid environment. Cultured oil was allowed to incubate at 37 °C for 16–18 h. Inhibition zone diameters were measured and their growth was evaluated.

## 3. Results and discussion

### 3.1. UV-Vis analysis of produced ZnO NPs

**Figure 4:**
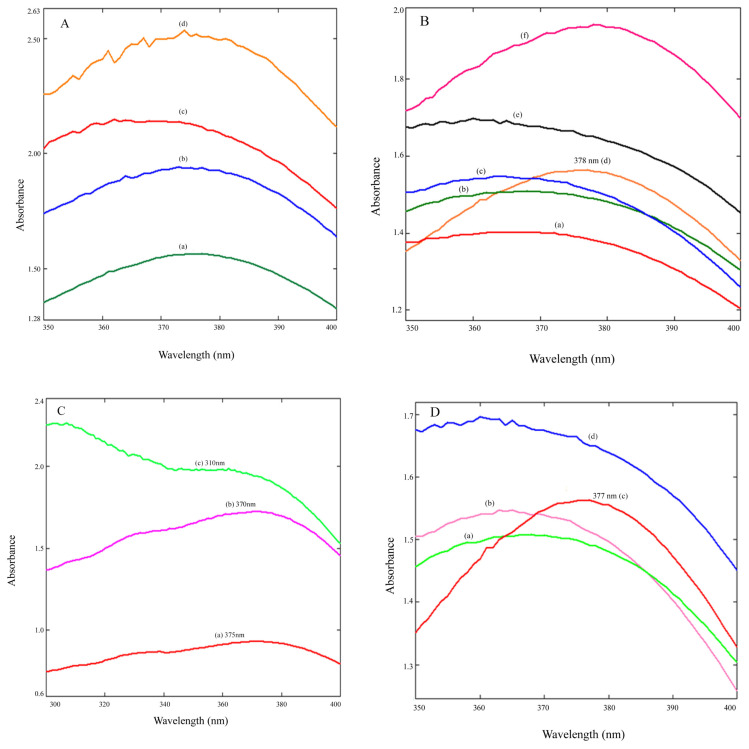
UV-Vis spectra of ZnO NPs; (**A**) Effect of time (a) 120, (b) 90, (c) 60, (d) 30 min; (**B**) Effect of ratio of Zn-Ac to CCF extract (a) 5, (b) 1.66, (c) 2.5, (d) 1.25, (e) 1, (f) 0.8; (**C**) Effect of temperature (a) 40 °C, (b) 60 °C, (c) 80 °C; (**D**) Effect of pH (a) 6, (b) 8, (c) 10, (d) 12.

**Figure 5:**
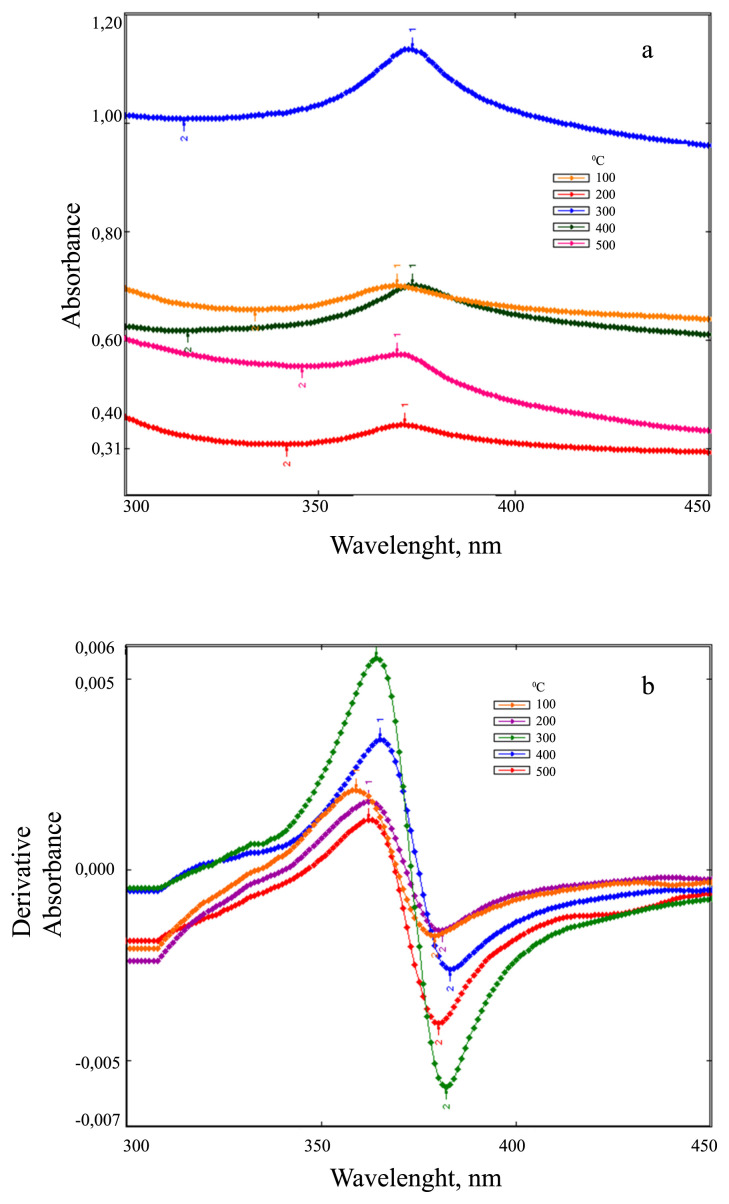
(a) Absorption and (b) first derivative absorption spectra of ZnO NPs produced at optimum conditions and calcined at 100 °C–500 °C.

In this study, we reported the synthesis of ZnO NPs using *Celosia cristata L*. flower extract for the first time. The abovementioned procedure (subsection 2.3) was followed at different time intervals (from 30 to 120 min) to optimize the time required to terminate the synthesis reaction. As seen in Figure 4a, it was determined that 120 min was sufficient to complete the reaction (λ_max_: 378nm). For optimization of volume ratio of Zn-Ac solution to CCF extract, synthesis procedure was repeated with different volume ratios (50/10, 50/20, 50/30, 50/40, 50/50 and 50/60 mL/mL; 5.0, 2.5, 1.66, 1.25, 1.0 and 0.8, respectively). As seen in Figure 4b, when 40 mL of CCF extract was used, a symmetrical and significant absorption peak occurred. This ratio (50/40, v/v) was chosen for the synthesis of stable ZnO NPs. The effect of temperature on the synthesis of ZnO NPs was investigated for three different temperatures (40 °C, 60 °C, and 80 °C). As the temperature increased, more than one maximum absorption peak was observed as a result of different particles formed. 40 °C was chosen as the appropriate temperature for ZnO NP synthesis (Figure 4c). When the effect of the environment pH on the synthesis of ZnO NPs was examined, (Figure 4d), an increase in absorbance values was observed when increasing the pH from 6 to 12. But a symmetric and significant peak at pH 10 (λ_max_: 378nm) showed that this pH value was suitable for the synthesis of stable ZnO NPs. Optimum operating conditions for green synthesis are; Zn-Ac/CCF extract ratio: 1.25, reaction temperature: 40 °C, reaction time: 120 min and pH: 10. ZnO NPs produced in different volume ratios of Zn-Ac solution were calcined at 100 °C–500 °C. Absorption and first derivative absorption spectra of these calcined nanoparticles were recorded in the wavelength range of 300–500 nm. For this purpose, 0.01 g of dried ZnO NPs were weighed and dissolved in 10 mL of ethyl alcohol. A total of 2 mL of this solution was taken and diluted to 10 mL with distilled water. The maximum wavelengths of these produced ZnO NPs are given in Table 3. Absorption and first derivative absorption spectra of ZnO NPs synthesized at optimum conditions and calcined at 100 °C –500 °C are shown in Figures 5a and 5b. We used the first derivative spectra to eliminate interferences and background effects from the colloidal solution and to better determine the peak maxima. The ZnO NPs produced at optimum conditions displayed symmetric and sharp peaks for all variations. When the temperature increases from 100 °C to 500 °C, the maximum wavelengths were seen at 357, 360, 364, 366, and 368 nm, respectively. The nanoparticles were stored in a desiccator in a dried form and found to be stable after 11 months of storage at room temperature.

### 3.2. FTIR spectra

**Figure 6:**
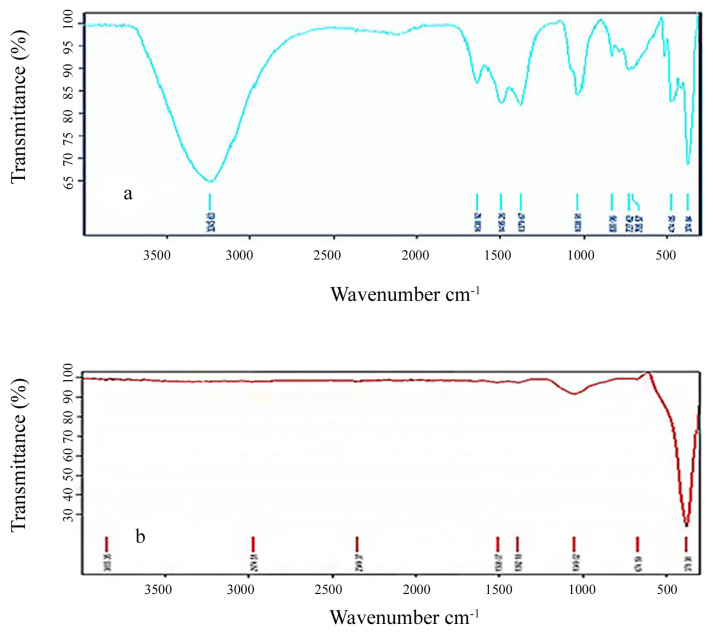
FTIR images of (a) the aqueous extract of CC flowers (b) the produced ZnO NPs at the optimum conditions and calcined at 300 °C.

To see the role of the CCF aqueous extract in reduction and to confirm the synthesis of the ZnO NPs, FTIR spectra of CCF aqueous extract and the ZnO NPs obtained under optimum conditions and calcined at 300 °C were recorded in the range from 400 to 4000 cm^−1^. The FTIR band positions of CCF extract are at 3245, 1640, 1495, 1379, 1040, 727, 474, and 375 cm^−1^ (Figure 6a). The broad and strong band centered at about 3245 cm^−1^ is the characteristic –OH stretching vibrations. The 1640 cm^−1^ peak results from the stretching bands of C‚O functional groups [40]. As seen in Figure 6b, the ZnO NPs spectrum was dominated by the band of 381 cm^−1^ that can be ascribed to the stretching of the Zn-O band [41]. This band confirmed the formation of ZnO NPs. The results are similar to other literature findings [42, 43]. Calcination was found to significantly reduce carboxylate and hydroxyl impurities in the CCF extract. The loss of spectral peaks of carboxylate impurities indicates that the zinc carboxylate decomposes and converts to ZnO during calcination. The hydroxyl peak follows a similar trend as shown in Figure 6b. These structural changes in FTIR spectra show that the molecules in the plant extract are involved in the formation and stabilization of the ZnONPs.

### 3.3. SEM and TEM studies

**Figure 7:**
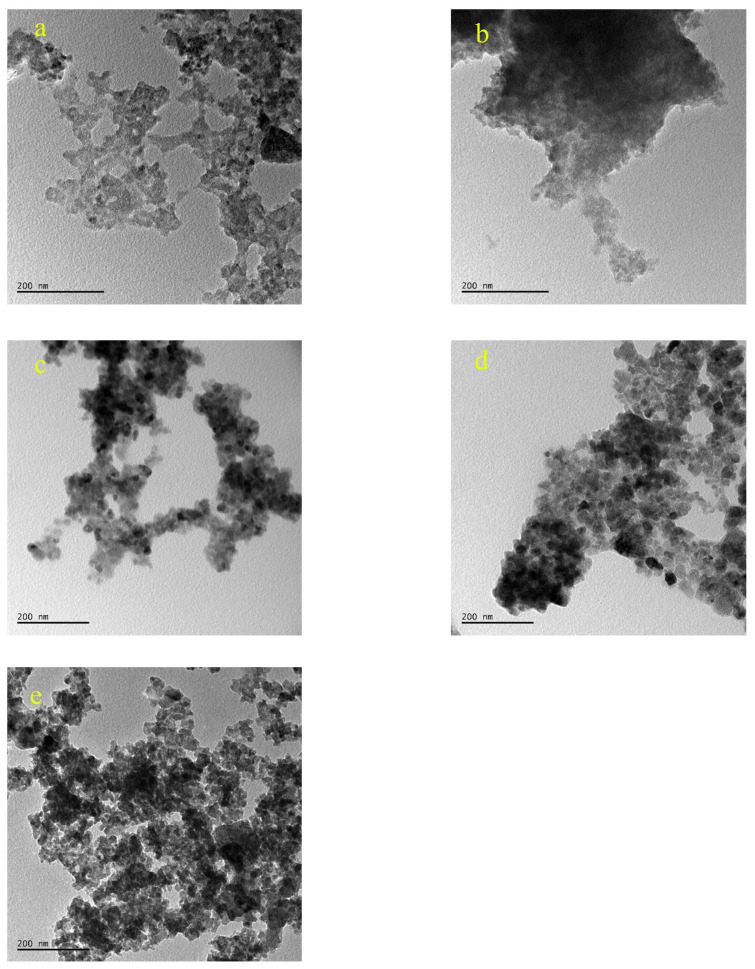
TEM images of ZnO NPs obtained at different calcination temperatures (a: 100 °C, b: 200 °C, c: 300 °C, d: 400 °C, e: 500 °C).

**Figure 8:**
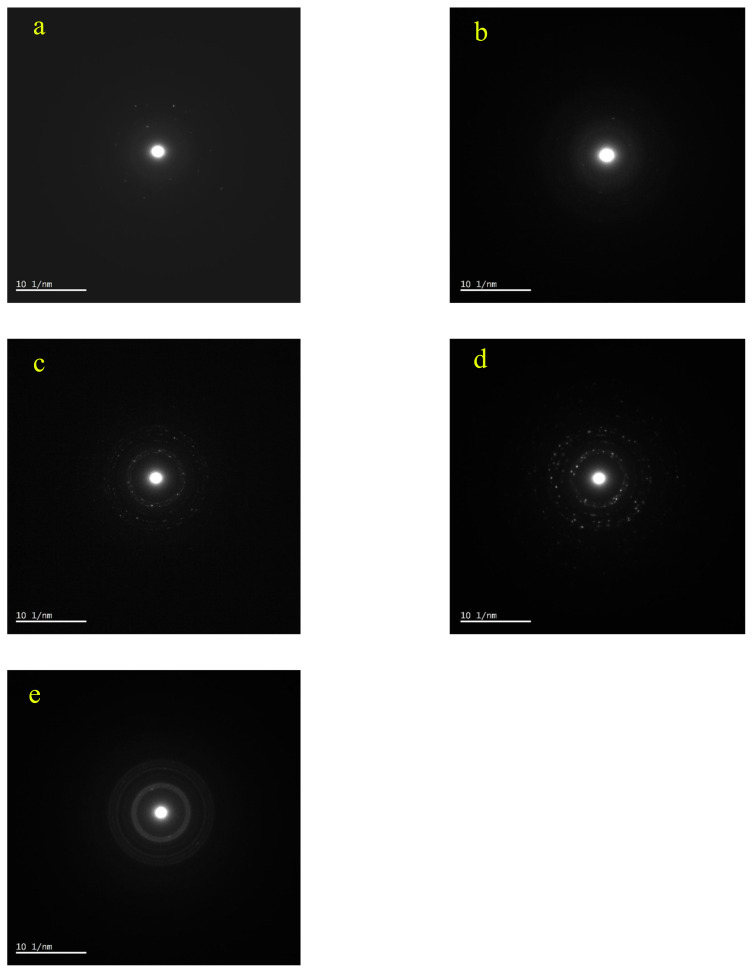
SAED patterns of TEM images given in Figure 7 (a: 100 °C, b: 200 °C, c: 300 °C, d: 400 °C, e: 500 °C).

Low magnification TEM images (Figures not shown) show that as the temperature increases the agglomeration a decreases and the structure is getting more homogenous. Moreover, as can be seen in Figures 7a–7e, there is a trend of decrease in nanoparticle size as the sintering temperature increases. Similarly, selected area electron diffraction (SAED) patterns show a few spots in low-temperature processes samples (Figures 8a and 8b). However, as the temperature increases first, monocrystalline spots are seen (Figures 8c and 8d) and finally in the 500 °C sample, the diffraction pattern shows solid rings (Figure 8e). Therefore from TEM investigations it can be stated that as the temperature increases, the crystallinity increases, and the average diameter of the nanoparticles decreases.

**Figure 9:**
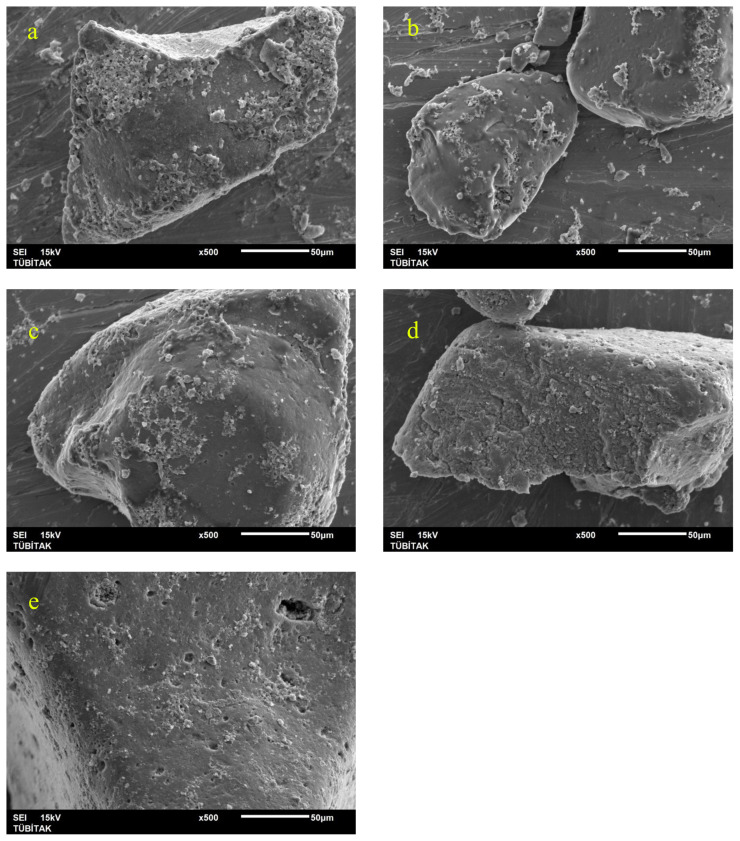
Low magnification SEM images (a: 100 °C, b: 200 °C, c: 300 °C, d: 400 °C, e: 500 °C).

**Figure 10:**
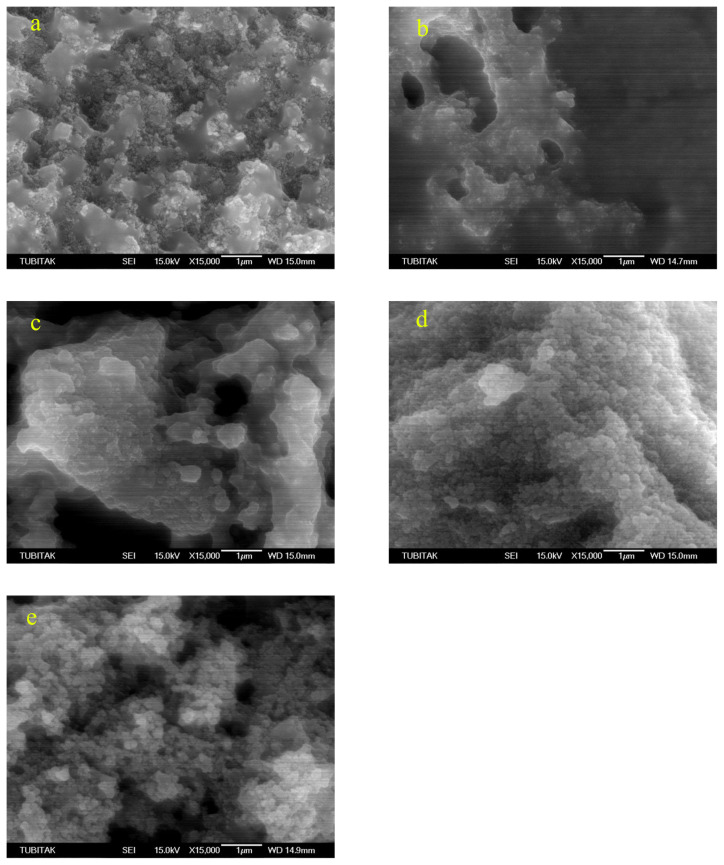
High magnification SEM images (a: 100 °C, b: 200 °C, c: 300 °C, d: 400 °C, e: 500 °C).

SEM images (Figures 9a–9e and 10a–10e) also show that at low temperatures the morphology is mostly mixed of flat and porous type. As the temperature increases the microstructure is more even and mostly nanoparticles are observed.

**Figure 11:**
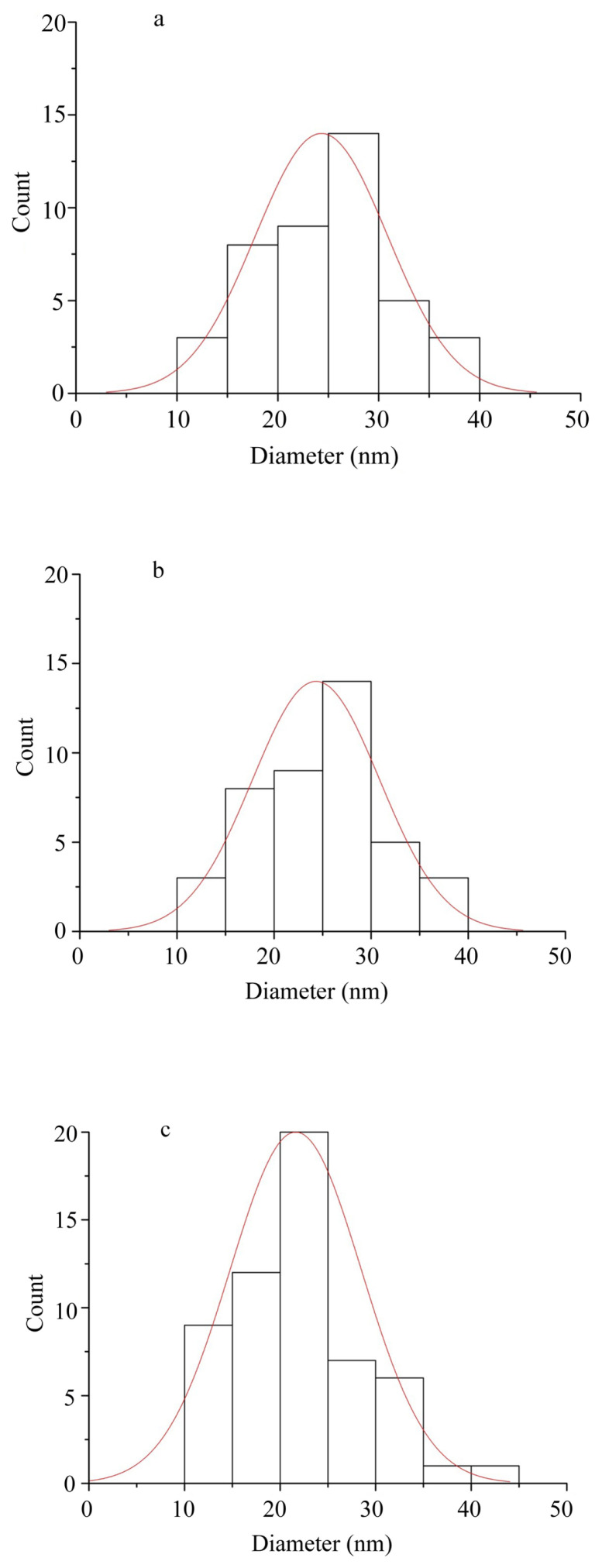
Histogram of dimension distribution of ZnO NPs produced at a) 300 °C, b) 400 °C, and c) 500 °C.

By disregarding big sized (>50 nm) and very small (<10 nm) particles from TEM, average particle size was determined as 24 nm for ZnO NPs produced at 300 °C, 27 nm for ZnO NPs produced at 400 °C and 22 nm for ZnO NPs produced at 500 °C by counting more than 40 particles for each sample. For ZnO NPs produced at 100 °C and ZnO NPs produced at 200 °C it was not possible to calculate average particle size since the samples showed mostly amorphous features. However, some nanosized particles were observed between 20 and 80 nm and some small particles were as small as 10 nm. It can be speculated that all samples had similar-sized nanoparticles that could be observed and measured with the help of TEM. Since there was agglomeration in the samples and since TEM gives only brief information about the average particle size; it is hard to give the exact average particle size (Figures 11a–11c).

### 3.4. Removal of BY28 and BV39 dyestuff and photocatalysis reaction mechanism

**Figure 12:**
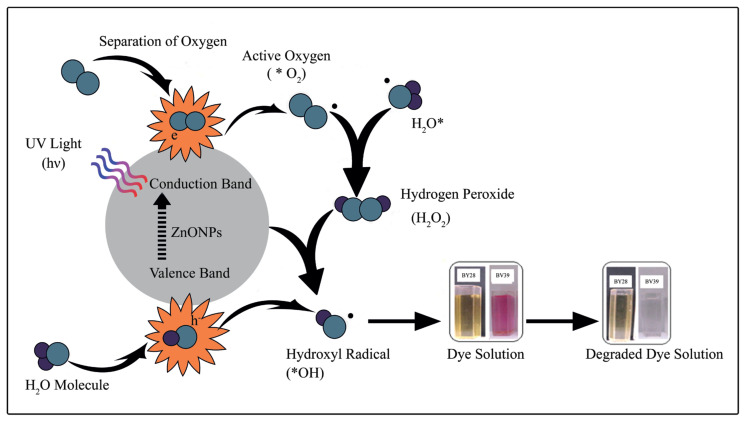
Schematic representation of photo catalytic degradation process.

ZnO NPs have been progressively researched for their photocatalytic implementation through photo-assisted oxidation, which is contemplated to be an encouraging arrangement to decontaminate biodegradable and organic contaminants from wastewater. The photocatalytic efficiency of ZnO NPs was evaluated by measuring the decolorization of the BV39 and BY28 dye solutions under UV light irradiation. Possible photocatalytic reaction under UV light irradiation using ZnO NPs as photocatalyst is as shown in Figure 12. Figure 12 describes the basis of the photocatalysis response procedure that happens through a series of reactions on the surface of ZnO NPs. The mechanism of photocatalytic response can generally be examined in four steps as photoexcitation, charge separation, displacement, and surface oxidation-reduction reactions. The ZnO photocatalysis can be irradiated by placing ZnO NPs suspension under natural sunlight and UV light. ZnO nanoparticles have the ability to produce electron hole (e–/h+) pairs when excited by photons with higher energy than their bandgap values under light. ZnO NPs whose surfaces are initially stimulated tend to migrate to the conduction band (CB) of an electron in the valence band (VB) and then create a hole in the valence band, thanks to this radiation on them. The excited electron (e–) will be transferred from the VB to the CB of the semiconductor by absorbing UV light energy higher than its energy gab thus, generates the (e^−^/h^+^) pair. The (e^−^/h^+^) combine can be either reunite with each other or a series of photoreactions can be initiated as shown in Figure 12. The holes or electrons that are formed in this way can directly oxidize the dyestuff molecules. Or, the oxidation of dyestuff molecules takes place through water or OH radicals generated from the oxidation of OH^−^ by the h^+^, or oxygen radicals (O_2_•,O•) formed from the oxidation of oxygen absorbed at the catalyst surface. The produced electron hole (e^−^/h^+^) pairs migrate to the surface of ZnO NPs, thereby creating redox reactions and serving the formation of reactive oxygen species. Holes act as oxidizing agents and react with water and OH^−^ ions to form hydroxyl radicals (OH•). The electrons in the CB have the status of reducing agents and are responsible for reducing the oxygen adsorbed on the surface of the ZnO nanophotocatalyst and generating active radicals and creating O^2−^•. All these produced free radicals, and especially the OH•, attack the organic pollutants that are physically attached to the ZnO surface where photocatalytic degradation occurs and which are stimulated by light, rapidly converting them into harmless compounds such as CO_2_ and H_2_O [24, 44].

#### Creating standard curve equations

When the UV-Vis spectrophotometric method was applied to the determination of dyestuffcon centration in the reaction mixture, higher values were found due to turbidity. Since the dye concentration in turbid samples cannot be determined directly from absorbance measurements, the first derivative method was applied to calculate the BY and BV dye concentration of the solution to overcome this difficulty. This makes the derivative method particularly useful for quantitative determination of the dyestuff in the presence of turbidity or when the background absorbance is high or not very well detailed.

Increasing concentrations of dyestuff solutions were prepared (3.0–15.0 μg/mL for BY28 and 3.0–15.0 μg/mL for BV39) and the first derivative absorption spectra of the prepared standard solutions were recorded between 300 and 650 nm against distilled water. In the spectra recorded for the BV39 dyestuff solutions, the peak amplitudes (_1_D values) were measured at 571 nm for the determination of BV39 and in the spectra recorded for the BY28 dyestuff solutions, the peak amplitudes (_1_D values) were measured at 400 nm and 474 nm for the determination of BY28. Calibration graphs for BV39 and BY28 were obtained with straight lines between 3.0 and 15.0 μg/mL concentration range. Standard curve equations (Equations 1 and 2) were used to calculate the concentration of dyestuff.


(1) Standard curve equation created for BV39: D1571=0.0464C+0.0066 (R2=0.9991)

(2) Standard curve equation generated for BY28: D1401-474=0.0215C+0.0119 (R2=0.9975)

The photocatalytic efficiency symbolized by the color removal was calculated based on Equation (3).


(3) $$\text{Percentage of dyestuff decolorization:}\;(\text{DD}\%)=[({\text{C}}_{0}-{\text{C}}_{\text{t}})/{\text{C}}_{0}]\times 100$$

Here, C_0_ is the initial concentration (μg/mL) of dyestuff solution and C_t_ is the concentration at different UV irradiation times.

**Figure 13:**
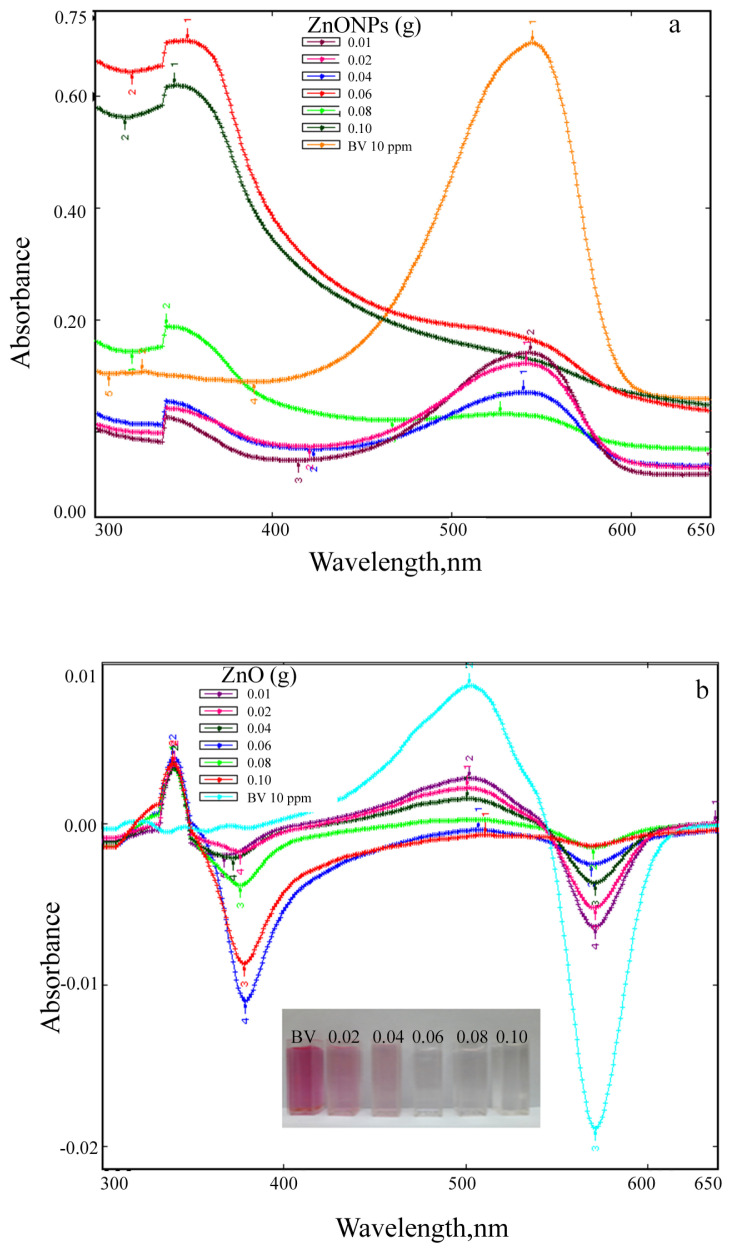
(a) Absorption and (b) first derivative absorption spectra taken after stirred different amounts of ZnO NPs with 10 ppm of BV39 solution under UV irradiation for 160 min (inset; visual of observed color change).

**Figure 14:**
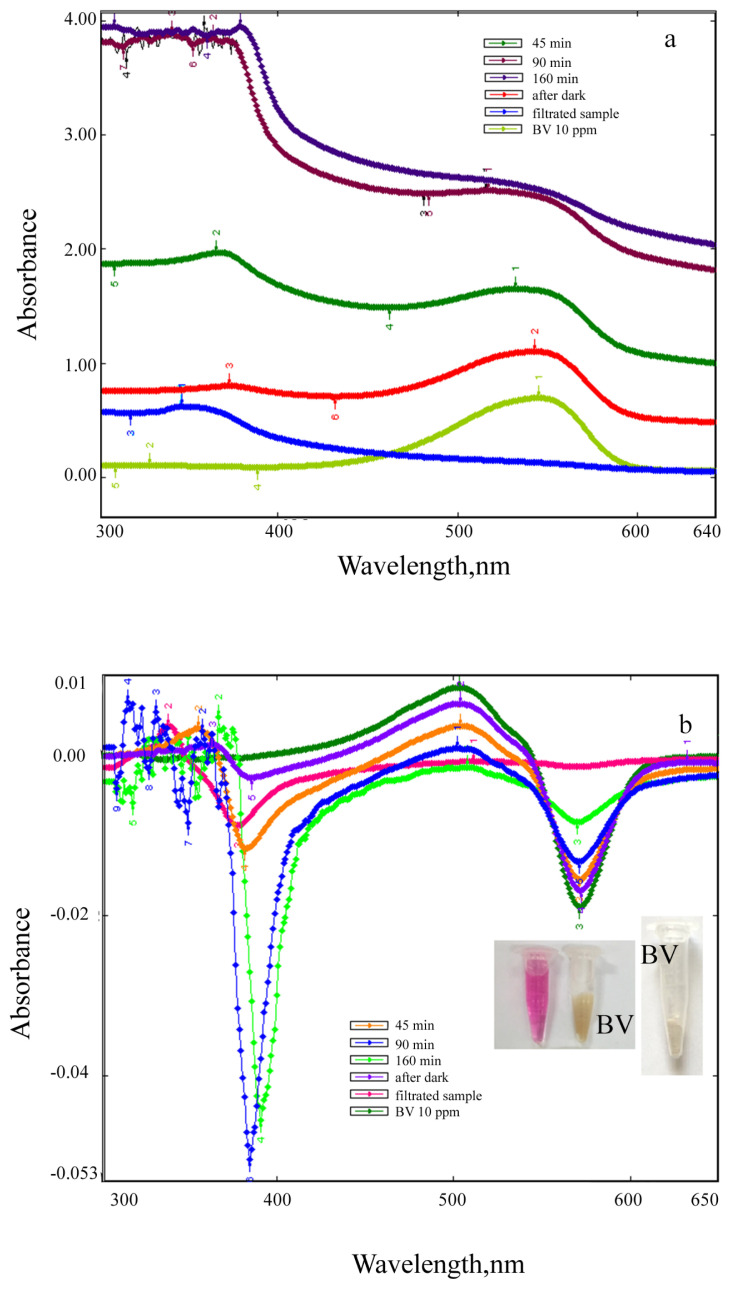
(a) Absorption and (b) first derivative absorption spectra recorded after stirred constant amount (0.1 g) of ZnO NPs with 10 ppm of BV39 solution under UV irradiation for different times (inset; visual of observed color change).

Absorption and first derivative absorption spectra taken after stirred different amounts of ZnO NPs with 10 ppm of BV39 solution under UV irradiation for 160 min are given in Figures 13a and 13b. Absorption and first derivative absorption spectra recorded after stirred constant amount (0.1 g) of ZnO NPs with 10 ppm of BV39 solution under UV irradiation for different times are presented Figures 14a and 14b. Since the background absorbance is high as seen in Figures 13a and 14a, the dyestuff concentration cannot be determined directly from absorbance measurements. The dye concentration in the solutions was accurately determined by taking the derivative spectra of the centrifuged and filtered solutions (Figures 13b and 14b).

When increasing amounts of ZnO NPs (0.01, 0.02, 0.04, 0.06, 0.08, and 0.1 g) were added to the individually prepared dyestuff solutions (at a concentration of 10 μg/mL), it was observed that the peak intensity decreased and the color of the solution decolorized (Figure 13b). It was observed that BV39 dyestuff was very slightly adsorbed to ZnO NPs, but the color of the solution faded over time as a result of photocatalytic degradation (Figure 14b). After 160 min, BV39 was successfully decolorized up to 95%. The color changed from pink to transparent. Decolorization of 10 μg/mL of BV39 dyestuff under operating conditions at room temperature was achieved in 160 min with 0.06 g of ZnO NP. As seen in Figures 13 and 14, the peaks seen at the spectra of ZnO NPs + dye solutions, which are not seen in the spectra of dyestuff solutions in the 300–400 nm wavelength range at both absorption and derivative spectra, were attributed to the interaction of ZnO NPs with the dyestuff due to photocatalysis. It is seen at the absorption and derivative absorption spectra that this interaction, which is less in the dark, accelerates under UV light. After 160 min irradiation under UV light, the ZnO NPs were separated from the reaction mixture by centrifugation and filtration. The peaks seen at 345 nm and 367 nm respectively in the absorption and derivative absorption spectra of the filtrate and the slightly colored (almost colorless) ZnO NPs indicate that ZnO NPs successfully decomposed the BV39 dyestuff, resulting in less toxic CO_2_ and H_2_O products. Due to the color removal, there is a decrease in the intensity of the derivative absorption peaks giving a maximum at 502 and 571 nm. Measurements taken at both maximum wavelengths can be used to accurately calculate color removal.

**Figure 15:**
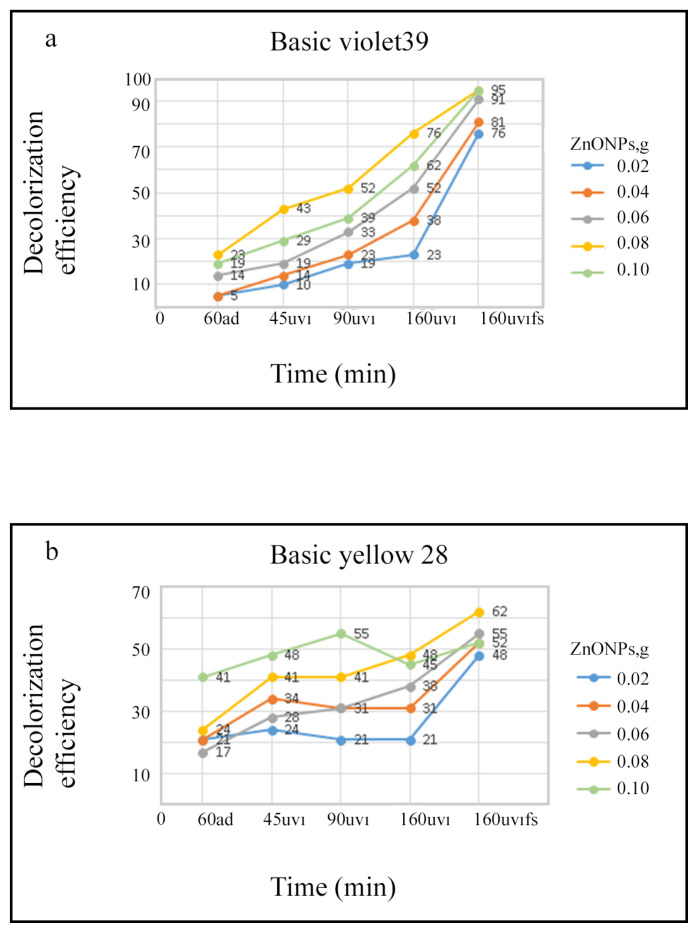
Graphical representation of the percentage of color removal of (a) BV39 and (b) BY28 dyestuff in the presence of different amounts of ZnO NPs photocatalyst as a function of irradiation under UV light for different times (ad: after dark, uvı: ultraviolet irradiation, uvıfs: filtered sample).

Graphical representation of the percentage of color removal of BV39 and BY28 dyestuff in the presence of different amounts of ZnO NPs photocatalyst as a function of irradiation under UV light for different times are given in Figures 15a and 15b. Decolorization of BV39 solution, which is stirred for 1 hour in the dark, is 5%–23%, depending on the amount of ZnO NPs. It is seen that as the amount of ZnO NPs increases, the color removal increases (Figure 15a). For this dyestuff solution after 160 min irradiation under UV light, 76%–95% decoloring was observed depending on the amount of ZnO NPs (from 0.02 to 0.1).

**Figure 16:**
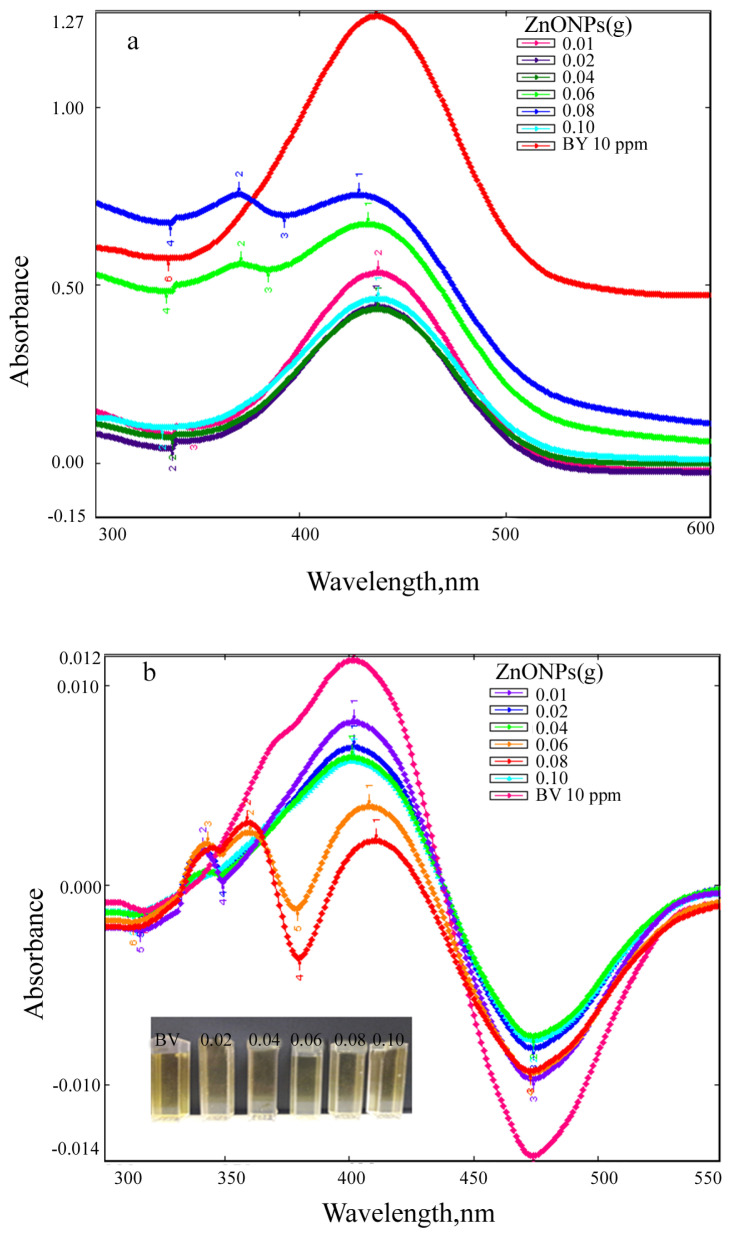
(a) Absorption and (b) first derivative absorption spectra taken after stirred different amounts of ZnO NPs with 10 ppm of BY28 solution under UV irradiation for 160 min (inset; visual of observed color change).

**Figure 17:**
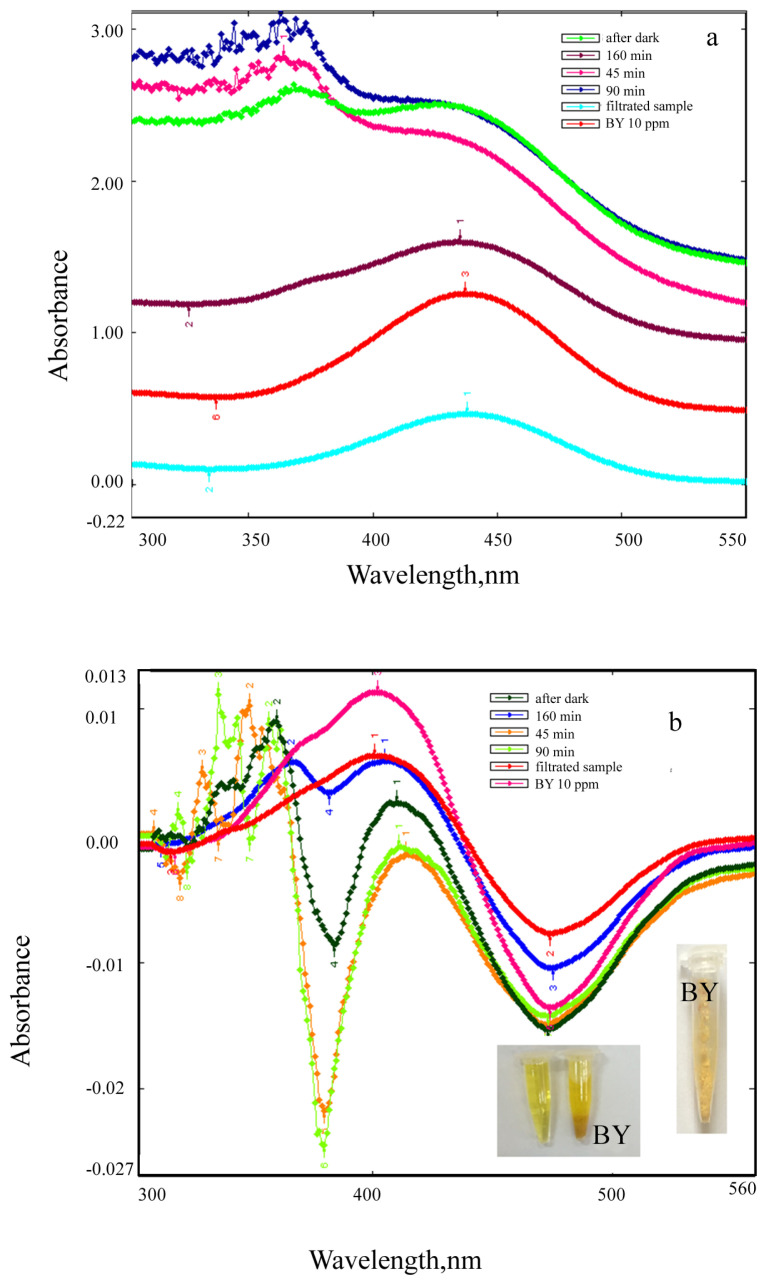
(a) Absorption and (b) first derivative absorption spectra recorded after stirred constant amount (0.1 g) of ZnO NPs with 10 ppm of BY28 solution under UV irradiation for different times (inset; visual of observed color change).

**Figure 18:**
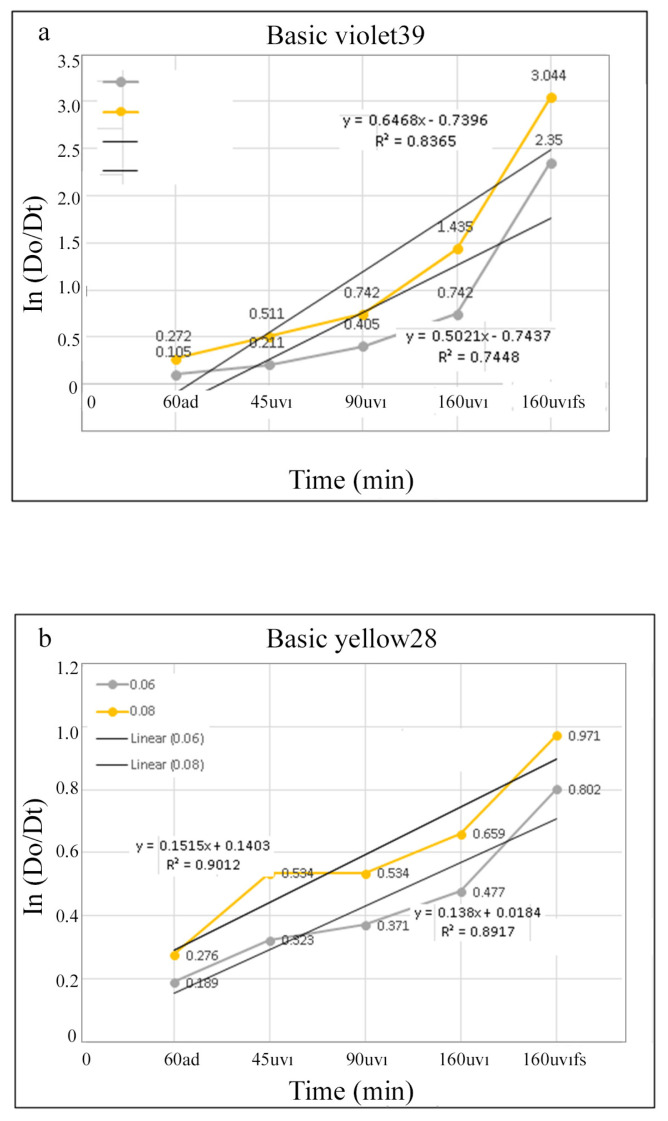
A graph demonstrating the correlation between ln(Do/Dt) and time for the first order photo catalytic degradation of (a) BV39 and (b) BY28 using ZnO NPs as a photo catalyst under UV light irradiation.

Absorption and first derivative absorption spectra taken after stirred different amounts of ZnO NPs with 10 ppm of BY28 solution under UV irradiation for 160 min are presented in Figures 16a and 16b. Absorption and first derivative absorption spectra recorded after stirred constant amount (0.1 g) of ZnO NPs with 10 ppm of BY28 solution under UV irradiation for different times are given in Figures 17a and 17b. When the derivative spectra taken during the photocatalysis process of the BY28 dyestuff are analyzed (Figures 16b and 17b), it is seen that the total derivative absorbance values (peak to peak) of the two peaks at 401 and 474 nm wavelengths should be evaluated. The color removal percentage of this dyestuff was calculated by using the total derivative absorbance values measured at both wavelengths. As seen in Figure 15b, it was observed that as the amount of ZnO NPs increased, the color removal increased and a large percentage of the color removal occurred in the dark. It was observed that the color removal increased from 17% to 41% depending on the increasing amount of ZnO NPs (from 0.02 g to 0.1 g). After 160 min irradiation under UV light, it was observed that the color removal increased to 48%–62% depending on the amount of ZnO NPs (Figure 15b). The absence of peaks in the 300–400 nm wavelength range in the absorption and derivative absorption spectra of the filtered samples after 160 min irradiation under UV light and the yellow color of ZnO NPs highlights the adsorption phenomenon in the photocatalysis reaction. Figure 18a represents a plot of ln(Do/Dt) against time and the spectra demonstrated a straight relationship against time. This displayed a pseudo first-order kinetics of the photodegradation of BV39 by ZnO NPs via UV irradiation. The rate constant (k) values calculated for the studies using 0.06 g and 0.08 g photocatalyst were 0.5021 and 0.6468, respectively, and the correlation coefficient (R^2^) values in the graphs were calculated as 0 .7448 and 0.8365. Correlation coefficient values lower than 0.9 supports the low adsorption rate.

In a similar way, Figure 18b represents a plot of ln(Do/Dt) as a function of response time for the degradation of BY28 by ZnO NPs. The rate constant values calculated for the studies using 0.06 g and 0.08 g ZnO NPs as photo catalyst were 0.138 and 0.1515, respectively, and the correlation coefficient values in the graphs were calculated as 0.8912 and 0.9012. In either case, the correlation coefficients are closer to 1, which gives the best adsorption rate. The findings support the adsorption phenomenon seen for BY28 dye. It was observed that decolorization of the dye molecules by the produced ZnO NPs follows the psedo first order kinetics. This finding is related to the findings of Neppolian et al. [45] who indicated that the photo degradation of mostorganic molecules is defined by the psedo first order kinetics. So, the kinetics of the degradation of dyestuff molecules can be demonstrated as follows:ln(D_0_/D_t_) = kt (4),

where k is the first order rate constant (min^−1^), D_t_ and D_0_ are the first derivative absorbance values at time t and t = 0, respectively.

The increase of the amount of catalyst can increase the degradation efficiency due to the greater amount of active surface area of the catalyst and higher light absorption. Increasing active surface area means more active sites for performing photocatalytic reactions and more catalyst are exposed to light. Thus, the amount of absorbed photons and degradation efficiency increase. However, when the amount of catalyst exceeds a certain level, light penetration will be reduced due to the increased amount of suspension. When the turbidity increases, light scattering will occur, leading to a less effective photoactive suspension volume. Another problem with excessive amount of photo catalyst is the formation of aggregation. When particles are collected, not all surfaces are exposed to irradiation. Thus, the absorption of photons will not increase at a geometric rate [46,47]. Thus, increasing the catalyst does not mean that there will be a linear increase in the decay rate. In this study, when 0.1 g ZnO NPs is used in the photocatalytic decolorization of BY28 dyestuff, the decreasing efficiency percentage can be explained by the information given above (Figure 15b).

The decolorization percentages for 10 ppm dyestuff solutions under UV light irradiation in 160 min without using ZnO NPs are 4.0% and 7.9% for BY28 and BV39, respectively. In this study, ZnO NPs have been used successfully for dyestuff decolorization. Some of the studies on photo catalytic activities of green synthesized ZnO NPs are given in Table 4. In the Table 4, a comparative study of our results with the others reported was made in terms of time and yield %. As seen, the findings are in agreement with other studies.

### 3.5. Investigation of antibacterial efficiency of ZnO NPs

In this study, the antimicrobial efficiency of both CCF extract and ZnO NPs which produced with different amounts of CCF extract and calcined at various temperatures (100, 200, 300, 400, and 500 °C) were studied by modified disc diffusion method. As seen in Table 5, antibacterial efficiency of ZnO NPs is higher than CCF extract and increases slightly as the calcination temperature increases. It was observed that the inhibition zone diameters are proportional to the calcined temperatures. As explained in the TEM and SEM results, there is a tendency to decrease in nanoparticle size as calcined temperatures increase, and it is observed that the crystallinity increases as the temperature increases. Moreover, as the sintering temperature increases, the average diameter of the nanoparticles decreases and the microstructure is more homogeny and more nanoparticles are observed. The data in Table 3 and the results of SEM and TEM support the findings that the antibacterial effect increases as the particle size decreases depending on the calcined temperature. Particle size and concentration of ZnO NPs take part an important role in antibacterial capability. Many studies have reported that antibacterial efficiency is directly related to the concentration of ZnO NPs, similarly, the yield depends on the particle dimension, but this dependence is also influenced by the concentration of NPs [52,53]. Smaller sizes of ZnO NPs can easily penetrate bacterial membranes due to their large interface areas, by that means increasing their antibacterial efficiency. Numerous studies to investigate the effect of particle dimension on the antibacterial activity have reported that smaller dimension (higher specific surface areas) ZnO NPs show the highest antibacterial efficiency [54,55]. As shown in Table 3, as the Zn-Ac/CCF extract ratio increases, larger particles are formed and a red shift in wavelength is observed. These findings support our results in Table 5. As the Zn-Ac/CCF extract ratio increased, it was observed that the zone diameters decreased due to the formation of larger particles. It is clearly seen in Table 5, that ZnO NPs inhibit the growth of both gram-negative and gram-positive bacteria, and that the antibacterial efficiency of ZnO NPs is higher than CCF extract. The inhibition zone diameters range from 11 to 16 mm for *S. aureus* and range from 9 to 13 mm for *E. coli*. These results presented that the produced ZnO NPs may be more effective against gram-positive pathogens such as *S. aureus*. The produced ZnO NPs had showed agreeable antibacterial effects because of increased specific surface area as the decreased particle dimension causing to increased particle surface reactivity. Similar findings in the literature support our results [56,57].

## 4. Conclusion

Among metal oxide nanoparticles, ZnO is recently shown as the material of the future due to its unique properties and extensive applications. The plant-mediated (biosynthesis, green synthesis, biogenic synthesis) synthesis of metal oxide nanoparticles is an encouraging alternative to the conventional procedures of physical and chemical synthesis. In this study, the green method that is easy, inexpensive, safe, and useful and does not harm the environment was chosen to produce nanoscale ZnO. The metabolites of *Celosia Cristata* flowers extract were used as a bioreducing and stabilizing agent and Zn (CH_3_COO)_2_ as the metal precursor to produce ZnO NPs. Produced ZnO NPs were well defined by UV-Vis absorption spectroscopy, FTIR spectroscopy, SEM and HR-TEM techniques. In this study, zinc oxide nanoparticles, biosynthesized at a lower temperature (40 °C), in a shorter time (2 h), and at a low calcination temperature (300 °C) using *Celosia Cristata* flower extract, are spherical, small particle size (22–27 nm) and stable for a long time (approximately 1 year). The green synthesis of ZnO NPs using CC flower extract and the use of these particles as photocatalysts in the decolorization of both BV39 and BY28 dyestuffs is the first study in this field. The photo catalytic reaction efficiency of ZnO nanoparticles under 160 min UV light irradiation was high and 95%–100% color removal for BV39 dyestuff was observed. High reaction efficiency in a short time showed that ZnO NPs had significant photocatalytic activity for this dye. For decolorization of BY28 dye, low reaction efficiency (around 62%) was observed and ZnO NPs were dyed due to adsorption. Produced ZnO NPs also displayed antibacterial activity against *S. aureus* and *E.coli* bacterial strains and are more effective against gram-positive pathogens. The findings displayed that the antibacterial activity of ZnO NPs is related to the particle size and morphology. Green-produced ZnO NPs could be an alternate instead of chemical antibacterial agents.

The method developed in this study could be an alternative to the studies on this subject and produced green ZnO NPs can be a good alternative in the biomedical, food, cosmetic and pharmaceutical industries and environmental applications. Developed procedure avoids utilization of harmful and poisonous solvents. This environmentally benign synthesis procedure could be a competitive alternative to the conventional chemical and physical techniques. This new ecofriendly synthesis approach is a useful technique suitable for large-scale commercial production. If the zinc oxide production method, which is an indispensable raw material of the cosmetics industry and a raw material that is imported to our country, is commercialized, it will contribute to the national economy. These ZnO NPs, produced with the green method, will find use in smart dye (self-cleaning, antibacterial, etc.) antiultraviolet fabric, smart textile products, wastewater treatment and many other areas. Since the average particle size of the synthesized ZnO NPs is below 40 nm, toxicity analysis of these particles must be done in order to be used safely in both the pharmaceutical and cosmetic and food industries.

Some suggestions we can give to researchers working on this subject from the results of the study we have done so far: the precurser/plant extract ratio should be at least 1.5–2.0, pH 8–10, and the production temperature should be 40–60 °C, and the calcination temperature should be at least 300 °C. In photocatalysis reactions, spectrophotometric measurements should be taken in centrifuged and filtered samples and more accurate results will be obtained when the derivative spectra are taken.

In our future researchs, we aim to produce metal-doped ZnO NPs in order to increase the photocatalysis reaction efficiency in daylight (natural environment) because it is less costly. In addition, we aim to produce spherical, nontoxic, antibacterial and antifungal effective metal-doped ZnONPs in 40–100 nm particle sizes that could be used in the cosmetic, food and pharmaceutical industries.

## Figures and Tables

**Table 1:** Reaction conditions used in the production of ZnO NPs using different plants and some data on the produced particles.

Biological substrate	Precurser	Research purpose	Reaction time and temperature	Thermal treatment	Average size (nm)	Shape	Reference
*Lycopersicon esculentum* *Citrus sinensis* *Citrus paradisi* *Citrus aurantifolia*	Nitrate	Photo catalytic degradation	60 min, 60 °C	400 °C, 1 h	9.01 12.55 19.66 11.39	Polyhedral	[21]
*Neem leaves*	Nitrate	Antioxidant, photoluminescent and photo catalytic properties	350 ± 10 °C, 4 min			Hexagonal plates, bullets, flower, prismatic tip, closed pine cone	[22]
*Stevia rebaudiana leaves*	Zincnitrate		2 h, 50 °C	60 °C, 4h	500–1.000	Flower-shape	[23]
*A. carnosus leaves*	Nitrate	Antibacterial, photo catalytic activities	60 °C	400 °C, 2h	20–40	Quasi-spherical	[24]
*P. niruri plant*	Nitrate	Photo catalytic activity	60 °C	400 °C, 2h	25.61	Quasi-spherical	[25]
*Aloe vera leaf*	ZnSO_4_	Antibacterial effect	3 h, 60 °C	80 °C in vacuum oven, 24 h	8–18	Spherical, oval, hexagonal	[26]
*C. citriodora plants*	Zn (NO_3_)_2_	Photo catalytic activity	80 °C, 48 h	Oven over night	64	Hexagonal wurtzite	[27]
*Brassica oleracea L (broccoli extract)*	ZnCl_2_·7H_2_O	Photo catalytic efficiency	First: 70°C for 20 min Second:80 °C for 6 h	450 °C	14	Partial spherical	[28]
*Rambutan (Nephelium lappaceum L.) peel*	Zn(NO_3_)_2_	Photo catalytic efficiency	First: 80 °C for 2 h Second:90 °C for 10 h	450 °C	24–40	Spherical shape	[29]
*C. fistula leaf*	Zn(NO_3_)_2_	Photo catalytic, antioxidant, antibacterial activities		400 °C, 10 min	5–15	Hexagonal wurtzite	[30]
*M. indica leave*	Zinc nitrate	Antioxidant and cytotoxic properties	Room temp, 6h dried 80 °C	450 °C	60	Nearly spherical and hexagonal	[31]
*Celosia Cristata*	Zn(CH_3_COO)_2_	Photo catalytic degradation antibacterial effect	40 °C, 120 min	300 °C, 2 h	22–27	Spherical shape	Current study

**Table 2:** Properties of the dyestuffs used in this study.

Dye properties 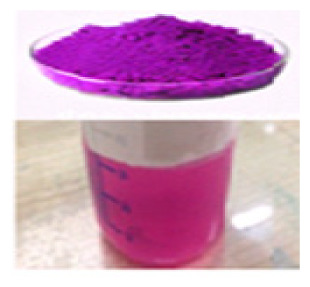 Bazik violet39	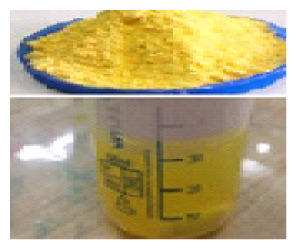 Basic yellow28
Chemical name: C.I. Basic violet 39	C.I. Basic yellow 28
Molecular structure: Xanthene class	Azomethine class
CAS number (s): 87912-74-1	54060-92-3
Maximum wavelength: 545 nm	440 nm

**Table 3:** Maximum wavelengths of ZnO NPs produced at different volume ratios of Zn-Ac solution to CCF extract and calcined at 100 °C–500 °C.

Calcination temperature	Volume ratio of Zn(CH_3_COO)_2_/CCF extract (v/v, mL/mL)				
(°C)	Wavelength (λ _max_, nm)				
	1.00	1.25	1.66	2.5	5.0
100	352	357	357	369	372
200	358	360	365	370	372
300	360	364	366	373	375
400	366	366	369	373	374
500	364	368	370	369	371

**Table 4:** Studies on photo catalytic activity of green synthesized ZnO NPs.

Reducing agent	Dye	Light type	Time (min)	Yield %	Reference
*Anisochilus Carnosus*	Methylene blue	UV light	90	~100	[24]
*Azadirachta indica*	Methylene blue	UV light	180	82	[48]
*Phyllanthus Niruri*	Methylene blue	UV light	30	~100	[25]
*Azadirachta indica*	Methylene blue	UV light	120	85	[22]
*Carissa Edulis*	Congo red	UV light	130	97	[49]
^1^ *Citrus Sinensis* ^2^ *Citrus Paradisi*	Methylene blue	UV light	180	^1^95 ^2^77	[21]
*Thymus Vulgaris*	Methylene blue	UV light	30	96	[50]
*Boswellia Mukul Gum*	Methylene blue	UV light	180	70	[51]
*Celosia cristata*	Basic violet 39	UV light	160	95	This study
*Celosia cristata*	Basic yellow 28	UV light	160	62	This study

**Table 5:** Inhibition zone diameters.

Samples (100 μL)	Calcination temperature (°C)	Inhibition zone (mm)	
		*S. aureus*	*E. coli*

CCF methanol extract		11	11

Zn-Ac/CCF extr: 1.25	100	13	10
	200	15	12
	300	16	13
	400	16	13
	500	16	13

Zn-Ac/CCF extr: 1.66	100	13	10
	200	14	12
	300	15	13
	400	15	13
	500	15	13

Zn-Ac/CCF extr: 2.5	100	11	9
	200	12	9
	300	13	10
	400	13	10
	500	13	10
